# The Number of Frames on ECG-Gated ^18^F-FDG Small Animal PET Has a Significant Impact on LV Systolic and Diastolic Functional Parameters

**DOI:** 10.1155/2021/4629459

**Published:** 2021-12-06

**Authors:** Christoph Eissler, Rudolf A. Werner, Paula Arias-Loza, Naoko Nose, Xinyu Chen, Martin G. Pomper, Steven P. Rowe, Constantin Lapa, Andreas K. Buck, Takahiro Higuchi

**Affiliations:** ^1^Department of Nuclear Medicine, University Hospital Würzburg, Würzburg, Germany; ^2^Comprehensive Heart Failure Center, University Hospital Würzburg, Würzburg, Germany; ^3^Graduate School of Medicine, Dentistry and Pharmaceutical Sciences, Okayama University, Okayama, Japan; ^4^Department of Nuclear Medicine, University Hospital Augsburg, Augsburg, Germany; ^5^The Russell H. Morgan Department of Radiology and Radiological Science, Johns Hopkins University School of Medicine, Baltimore, MD, USA

## Abstract

**Objectives:**

This study is aimed at investigating the impact of frame numbers in preclinical electrocardiogram- (ECG-) gated ^18^F-fluorodeoxyglucose (^18^F-FDG) positron emission tomography (PET) on systolic and diastolic left ventricular (LV) parameters in rats.

**Methods:**

^18^F-FDG PET imaging using a dedicated small animal PET system with list mode data acquisition and continuous ECG recording was performed in diabetic and control rats. The list-mode data was sorted and reconstructed with different numbers of frames (4, 8, 12, and 16) per cardiac cycle into tomographic images. Using an automatic ventricular edge detection software, left ventricular (LV) functional parameters, including ejection fraction (EF), end-diastolic (EDV), and end-systolic volume (ESV), were calculated. Diastolic variables (time to peak filling (TPF), first third mean filling rate (1/3 FR), and peak filling rate (PFR)) were also assessed.

**Results:**

Significant differences in multiple parameters were observed among the reconstructions with different frames per cardiac cycle. EDV significantly increased by numbers of frames (353.8 ± 57.7 *μ*l^∗^, 380.8 ± 57.2 *μ*l^∗^, 398.0 ± 63.1 *μ*l^∗^, and 444.8 ± 75.3 *μ*l at 4, 8, 12, and 16 frames, respectively; ^∗^*P* < 0.0001 vs. 16 frames), while systolic (EF) and diastolic (TPF, 1/3 FR and PFR) parameters were not significantly different between 12 and 16 frames. In addition, significant differences between diabetic and control animals in 1/3 FR and PFR in 16 frames per cardiac cycle were observed (*P* < 0.005), but not for 4, 8, and 12 frames.

**Conclusions:**

Using ECG-gated PET in rats, measurements of cardiac function are significantly affected by the frames per cardiac cycle. Therefore, if you are going to compare those functional parameters, a consistent number of frames should be used.

## 1. Introduction

Positron emission tomography (PET) can provide a reliable read-out of the myocardial glucose metabolism on a cellular level along with a concomitant assessment of cardiac systolic and diastolic function [1]. For instance, impaired diastolic function is often the earliest detectable sign of cardiac involvement in chronic heart disease, such as in patients afflicted with diabetic cardiomyopathy [2, 3], which renders PET as an attractive diagnostic modality for diabetes-related myocardial functional decline. Moreover, abnormal diastolic function is frequently detected in asymptomatic patients suffering from diseases such as systemic sclerosis, and therefore, the current status of diastolic function should be also carefully evaluated even in patients not suffering from primary cardiac damage [4–6]. For myocardial PET, myocardial gating is mandatory to sort the acquired data according to the phase of the cardiac cycle and provide systolic and diastolic function parameters in addition to a radiotracer-targeted interrogation of alterations on a cellular level [7].

In most cardiac PET examinations, electrocardiogram- (ECG-) gating is used, but there is no recommendation for a minimum number of frames per cardiac cycle to assess reliable values for LV functional parameters. Even though there have been studies investigating the impact of frame numbers in ECG-gated single photon emission computed tomography (SPECT) on the EF and LV volumes in humans [8, 9], data for small animals are still lacking. However, given their smaller heart size and higher heart rate relative to humans, small animal PET studies may require an increased number of frames per cardiac cycle to generate the required temporal resolution. Although ECG-gated ^18^F-FDG PET has been established for the assessment of LV volumes and LV ejection fraction (EF) in clinical [10–12] and preclinical PET studies [1, 13, 14], the influence of the chosen gating mode on the diastolic and systolic functional parameters in small animals has not been investigated yet.

Therefore, we aimed to evaluate the influence of the number of frames per cardiac cycle in an ECG-gated ^18^F-FDG PET study on the LV function parameters in both healthy and diabetic rats.

## 2. Material and Methods

### 2.1. Animal Model

All animal experimental protocols were approved by the regional Animal Care and Use Committee and performed according to the Guide for the Care and Use of Laboratory Animals (120/13) [15]. For this study, 12 rats of 13 weeks of age (ZL: Zucker Lean control (*n* = 6), ZDF: Zucker diabetic fatty (*n* = 6), Charles River) were included (mean body weight: 336 ± 61 g).

### 2.2. Small-Animal PET System and Imaging Protocol

A dedicated ring type, high resolution small animal PET scanner (Inveon micro PET, Siemens Medical Solutions Inc., Erlangen, Germany) was used for data acquisition, with specifications as previously described [16]. ^18^F-FDG was synthesized according to the manufacturer's instructions utilizing in-house cyclotron production of fluorine-18. Throughout the data acquisition, the animals were maintained under anaesthesia using 2% isoflurane after prolonged fasting (>10 h). To improve cardiac radiotracer uptake, approximately 37 MBq of ^18^F-FDG was injected via tail vein under hyperinsulinemic-euglycemic clamp conditions. As such, infusion of insulin (Insuman Rapid; Sanofi-Aventis, Paris, France) started at a rate of 240 mU/kg/min for 20 min followed by low-dose 12 mU/kg/min. To maintain euglycemia (blood glucose levels: 70–110 mg/l), varying rates of glucose (50.0% glucose solution) were added by checking blood glucose levels every 2 min and the intravenous radiotracer administration was established over a time period of at least 12 min. The list-mode PET data acquisition with continuous ECG recording was started shortly before injection. According to the ECG signals, the list-mode data of 15-35 min after radiotracer administration were sorted and histogrammed into 4-, 8-, 12-, and 16-frame sinograms for each animal and for each dynamic frame. The sinograms were reconstructed into tomographic images using a 2-dimensional ordered-subset expectation maximization method. A correction for ^18^F decay, random coincidences, and dead time was performed for all reconstructed images. Prior to the emission scan, a 13 min transmission scan was performed for attenuation correction.

### 2.3. PET Data Analysis

The generation of LV functional parameters was conducted using a dedicated automatic ventricular edge detection software (Heart Function View, Nihon Medi-Physics Co. Ltd., Tokyo, Japan) after the required corrections for the small rat heart size (5-fold magnification of the voxel size of the reconstructed images). The following parameters were calculated using a programme feature for gated myocardial perfusion SPECT: end-diastolic volume (EDV), end-systolic volume (ESV), left ventricular ejection fraction (EF, % of the stroke volume compared to the EDV), first-third ejection fraction (1/3 EF), peak filling rate (PFR, defined as the maximum dV/dt value divided by EDV, per second), first third mean filling rate (1/3 FR, defined as the average of dV/dt values in the first third of the filling time divided by EDV, per second), and the time to peak filling rate (TPF, defined as the time from end-systole to PFR, per millisecond). For further details, please refer to [1].

### 2.4. Statistical Analysis

All results are presented as the mean value ± standard deviation (SD). A *P* < 0.05 was considered statistically significant. Repeated measures of one-way ANOVA followed by Holm-Sidak's multiple comparison test were performed for the comparison of the differences between groups (GraphPad Prism 7, San Diego, CA).

## 3. Results

### 3.1. Stable Myocardial Radiotracer Retention Is Achieved 10 Min Post-Injection

**Figure 1:**
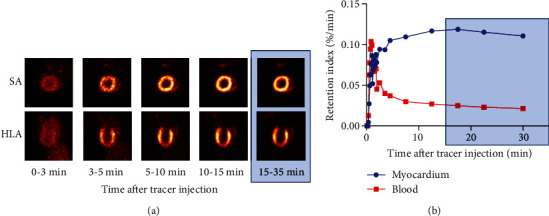
Dynamic ^18^F-FDG PET images (a) and time activity curve (b) after tracer administration via the tail vein under hyperinsulinemic-euglycemic clamp conditions. Frame data from 15-35 min (stable left ventricular delineation phase) were used for subsequent ECG-gating functional analysis. Dynamic PET imaging revealed a time course with rapid clearance of administered ^18^F-FDG from the blood pool (<10 min), followed by stable tracer accumulation in the myocardium (>10 min). SA: short axis; HLA: horizontal long axis.

Dynamic PET imaging revealed a time course with rapid clearance of administered ^18^F-FDG from the blood pool (<10 min), followed by stable radiotracer accumulation in the myocardium (>10 min) (Figure 1). Based on the dynamic radiotracer distribution pattern, a frame of 15-35 min after administration was used for the functional assessment with the automated left ventricular edge detection software.

**Figure 2:**
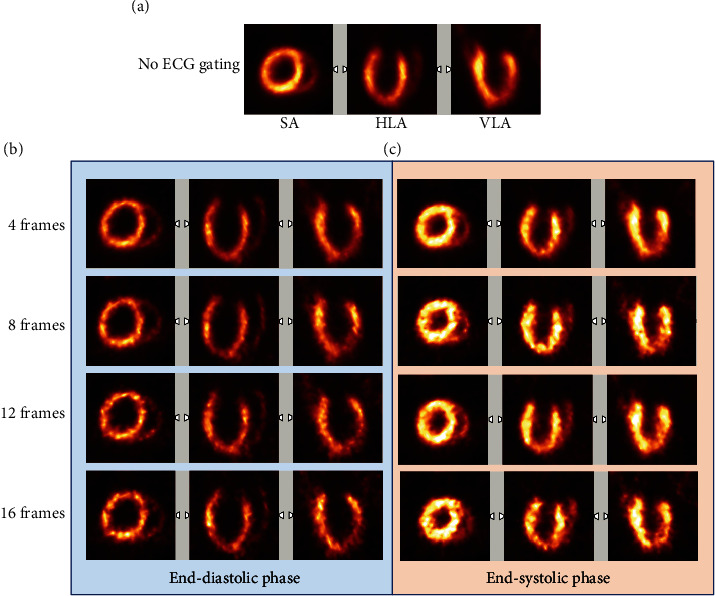
^18^F-FDG PET images of a rat heart with no ECG-gated reconstruction (a) and with different ECG-gated frame numbers at end-diastolic (b) and end-systolic phases (c). SA: short axis; HLA: horizontal long axis; VLA: vertical long axis.

Cardiac ECG-gated ^18^F-FDG PET images of all 12 animals were reconstructed. Due to the trade-off between high temporal resolution of gated PET images and reduced count density of the individual frames, the reconstructed cardiac images with high frame numbers per cardiac cycle showed increased noise (Figure 2).

### 3.2. Increased Number of ECG Frames Had a Significant Impact on All Investigated Systolic and Diastolic Functional Parameters

**Figure 3:**
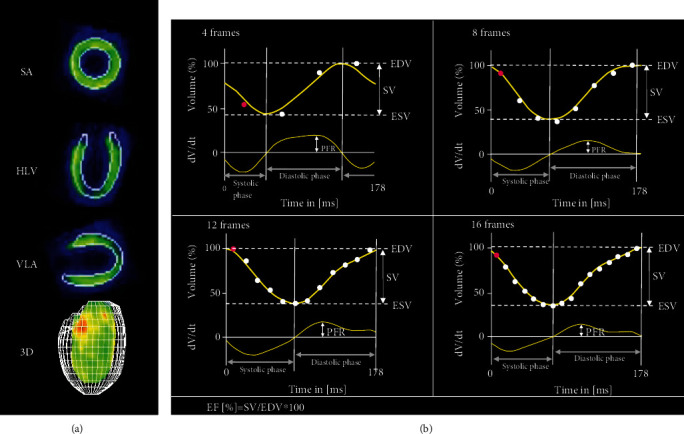
Example images of the edge detection of left ventricular wall and 3D images of the ventricular borders (a). Time-volume curves (b) and corresponding time-filling curve of the same animal with different numbers of gates per cardiac cycle. The smoothening of both curves due to the increasing number of volume values with increasing gate number is demonstrated as well as the calculation of the LV volumes and the PFR. EDV: end-diastolic volume; ESV: end-systolic volume; SV: stroke volume; EF: ejection fraction; PFR: peak filling rate.

**Figure 4:**
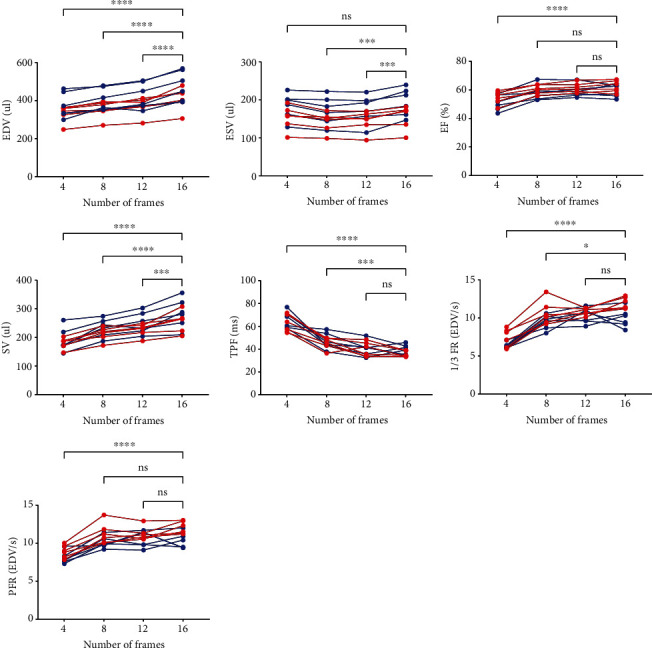
Comparison of the LV volumes and functional parameters of all animals (ZDF diabetic animal = blue, ZL controls = red) with different numbers of frames per cardiac cycle using Holm-Sidak's multiple comparison test with 16 frame data as reference. The LV volumes (EDV, ESV, and SV) of the 4, 8, and 12 frame data differ significantly from the reference, whereas for EF and diastolic parameters (TPF, 1/3 FR, and PFR), no significant difference between the reconstruction with 12 gates per cycle and the reference was recorded. EDV: end-diastolic volume; ESV: end-systolic volume; SV: stroke volume; EF: ejection fraction; TPF: time to peak filling; 1/3FR: first third filling rate; PFR: peak filling rate. ^∗,∗∗,∗∗∗,∗∗∗∗^Statistically significant vs. 16 frames per cardiac cycle (*P* < 0.05, *P* < 0.005, *P* < 0.0005, *P* < 0.0001).

Using the automatic left ventricular edge detection software, the left ventricular volume of each frame was measured to generate the left ventricular volume-time curve (Figure 3). Results of LV volume, systolic and diastolic parameters are summarized in Table 1 and Figure 4. An increase in the number of ECG frames led to an increase of the estimated LV volumes (EDV, ESV, and SV) and EF. In addition, diastolic parameters (TPF, 1/3 FR, and PFR) were also influenced by the number of frames. Of note, when comparing 12 frames to the reference of 16 frames, no significant differences could be recorded for TPF, 1/3 FR, and PFR.

### 3.3. Diastolic Functional Parameter Decrease in Diabetic, but Not in Control Rats

**Figure 5:**
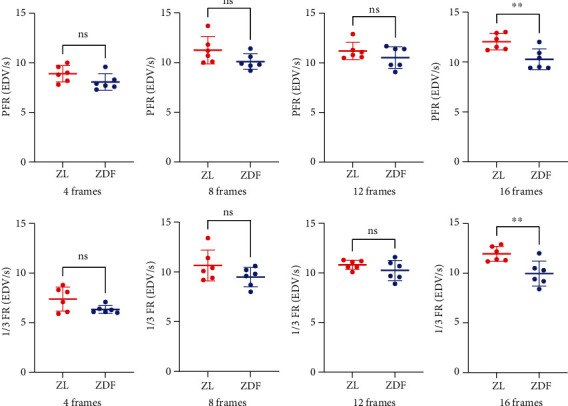
Impact of number of frames for diastolic parameters (PFR and 1/3 FR) between ZDF (diabetic rats) and ZL (control rats). For 16 frames per cardiac cycle, a significant difference was noted for both parameters (^∗∗^*P* < 0.005, far right), but not for 4, 8, and 12 frames per cycle.

When comparing diabetic (ZDF) and control (ZL) rats, significant differences of diastolic functional indices (PFR and 1/3 FR) were observed for 16-frame ECG gating (*P* < 0.005, respectively), while for other systolic and functional parameters, no significant differences were recorded. However, this did not apply for a lower number of frames (4, 8, and 12), neither for PFR nor for 1/3 FR (Figure 5).

## 4. Discussion

Regarding the influence of ECG-gating mode on LV parameters in rats, LV volumes and systolic parameters (EDV, ESV, and SV) significantly increased by the numbers of frames when compared to the highest number of applied frames. Systolic parameters (TPF, 1/3 FR and PFR), however, significantly differed between 4 and 16 frames. In addition, significant differences between diabetic and control animals in 1/3 FR and PFR in 16 frames per cardiac cycle were observed (*P* < 0.005), but not for 4, 8, and 12 frames. As such, in order to assess both systolic and diastolic parameters from cardiac ECG-gated ^18^F-FDG PET in rats, gating with an adequate number of frames per cardiac cycles seems to be necessary.

In clinical myocardial ECG-gated perfusion SPECT, a cardiac performance evaluation with 8 frames per cardiac cycle is most often performed, even though it has been shown that this approach can cause an overestimated ESV, underestimated EDV, and thereby an underestimated EF [8, 9, 17]. Moreover, in ECG-gated ^18^F-FDG PET studies, it has been demonstrated that reliable LV volumes and EF could be obtained by using 16 [18] and 8 frames [12] per cycle when compared to cardiac magnetic resonance imaging (MRI).

Even though the impact of the gating mode on the LV volumes in patients has previously been investigated, corresponding studies in small animals are still lacking. Small animal PET studies provide important insights into the pathophysiology of different diseases and alterations on a cellular level and may even allow monitoring of novel treatment approaches [19]. In this regard, ECG-gated micro-PET enables an observer-independent evaluation of both cardiac systolic and diastolic function in small animals [1, 9]. In the present study with rats using a dedicated micro-PET system, the values of the different reconstructions were all compared to the reconstructions with 16 frames per cardiac cycle, with the latter providing the highest temporal resolution. Except for ESV, the calculated parameters from the reconstruction with 4 frames per cardiac cycle showed a significant difference of all LV volumes and functional parameters. Moreover, the calculated TPF and 1/3 FR also differed significantly in the reconstructions with 8 frames compared to the reconstruction with 16 frames per cardiac cycle. Kurisu et al. [20] also demonstrated that in ECG-gated myocardial perfusion SPECT in human patients, a reconstruction with 8 frames per cardiac cycle resulted in an underestimated PFR compared to the 16-gate reconstruction. As such, given the present findings in rats, a reconstruction with 4- or 8- frames per cardiac cycles may not be adequate to achieve the necessary temporal resolution for both human and small animal ECG-gated PET studies. Such information may be of value, e.g., for planning translational studies from rodents to humans in nuclear cardiology [19].

Unfortunately, it is not possible to increase the number of cardiac frames per cardiac cycle since the count density per cardiac gate declines with an increased number of frames and, therefore, leads to a prolonged scan time or higher administered activity [7]. Therefore, the optimal number of frames per cardiac cycle may be of importance for an accurate assessment of LV parameters along with a shorter scan time and smaller amount of the injected radiopharmaceutical, which further reduces the distress for the animals and also allows for an increased experimental throughput in a small animal PET lab. Moreover, in line with the present rat study demonstrating that gating with an adequate number of frames per cardiac cycles is necessary, previous experimental approaches investigating mice yielded comparable results. For instance, Stegger et al. [14] also used 16 frames per cardiac cycle in a murine ^18^F-FDG PET study and demonstrated a significant overestimation of the EDV but a similar EF relative to cardiac MRI. Brunner et al. [21] evaluated the function via ^18^F-FDG PET with only 8 frames per cardiac cycle in a murine model of dilated and ischemic cardiomyopathy and reported a significant underestimation of the EF and significant overestimation of both the ESV and the EDV in all groups. Taken together, these findings support the notion that for a precise assessment of LV parameters in small animal PET studies, an appropriate number of frames per cardiac cycle are needed, and the number of frames should be consistent if you are going to compare those value.

There are several limitations to this study. We investigated influence of ECG-gated frames per cardiac cycle and were not able to comprehensively study the other parameters that might affect the LV functional parameters by ECG-gating PET: for example, the differences in image acquisition time, tracer injection dose, and imaging scanner. Relatively standard acquisition condition and widely available PET tracer ^18^F-FDG are used in this study, except for varying the number of frames per cardiac cycles, and further studies might be needed for each of the exceptional protocols.

## 5. Conclusions

We performed ECG-gated ^18^F-FDG PET in healthy and diabetic rats to acquire images with 4, 8, 12, and 16 frames per cardiac cycle and quantified them to calculate LV volumes and systolic and diastolic parameters. Our data suggest that an adequate high number of frames per cardiac cycles are necessary for the estimation of systolic and diastolic parameters. These findings corroborate previous reports in humans and, therefore, may be of relevance for planning translational studies in nuclear cardiology.

### 5.1. New Knowledge Gained

In rats, the number of frames has a significant impact on the calculation of both systolic and diastolic LV functional parameters. An increased number of frames per cardiac cycle may provide a better evaluation of the LV function in rats, which may be of relevance for planning preclinical studies in nuclear cardiology.

## Figures and Tables

**Table 1:** Comparison of the LV functional parameters between the different gated reconstructions of all rats (*n* = 12).

No. of frames	4	8	12	16
*Left ventricular volumes and systolic parameters*				
EDV (*μ*l)	353.8 ± 57.7^∗∗∗∗^	380.3 ± 57.2^∗∗∗∗^	398.0 ± 63.1^∗∗∗∗^	444.8 ± 75.3
ESV (*μ*l)	168.3 ± 35.3	156.9 ± 34.5^∗∗∗^	159.1 ± 35.2^∗∗∗^	174.6 ± 38.4
SV (*μ*l)	185.5 ± 31.6^∗∗∗∗^	223.5 ± 28.9^∗∗∗∗^	238.9 ± 32.0^∗∗∗^	270.2 ± 45.1
EF (%)	52.7 ± 5.1^∗∗∗∗^	59.1 ± 4.3	60.4 ± 3.9	61.0 ± 4.4
*Left ventricular diastolic parameters*				
TPF (ms)	63.9 ± 7.5^∗∗∗∗^	46.2 ± 6.0^∗∗∗^	40.3 ± 6.2	37.7 ± 4.2
1/3 FR (EDV/s)	6.9 ± 1.0^∗∗∗∗^	10.1 ± 1.4^∗^	10.5 ± 0.8	11.0 ± 1.4
PFR (EDV/s)	8.5 ± 0.9^∗∗∗∗^	10.7 ± 1.2	10.9 ± 1.0	11.2 ± 1.3

Data are presented as the mean values ± SD. EDV: end-diastolic volume; ESV: end-systolic volume; SV: stroke volume; EF: ejection fraction; TPF: time to peak filling; 1/3FR: first third filling rate; PFR: peak filling rate. ^∗,∗∗,∗∗∗,∗∗∗∗^Statistically significant vs. 16 frames per cardiac cycle (*P* < 0.05, *P* < 0.005, *P* < 0.0005, *P* < 0.0001).

## Data Availability

The datasets generated and/or analyzed during the current study are available from the corresponding author on reasonable request.
